# ‘It Makes Me Feel Confident’: A Community Based Participatory Study of Experiences of Employment for People With Intellectual Disabilities

**DOI:** 10.1111/jar.70310

**Published:** 2026-10-18

**Authors:** Deanna Gallichan, Chloe Deacon, Karen Gresty, Simon Rundle

**Affiliations:** ^1^ University of Plymouth Plymouth UK; ^2^ Ivybridge Brewing Company C.I.C Ivybridge Devon UK

## Abstract

**Background:**

A social enterprise brewery requested help from researchers to understand their employment opportunities for people with intellectual disabilities (‘trainees’). A community‐based participatory framework (CBPR) was used to develop the research.

**Method:**

Digital storytelling was selected to enable trainees to express their views about their work. Six participants created digital stories with support from researchers. Transcripts of the stories were analysed using reflexive thematic analysis.

**Findings:**

Three main themes were developed: ‘Learning new skills’; ‘We do good work’; and ‘I belong at the brewery’, each with several subthemes. One overarching theme, ‘I feel more confident’ was developed to represent the ways in which different aspects of the work had developed their confidence.

**Conclusion:**

Working within the brewery seemed to have both immediate and far‐reaching benefits for participants. Further research would be needed to better understand the wider impact on the local community.

## Lay Summary

- A brewery employed people with learning disabilities. People were trained in brewing beer and serving customers. The brewery wanted to understand what people thought and felt about working there, and whether it had led to any changes in their lives.
- The brewery asked a local researcher for help. The researcher and the brewery worked together to decide how to do the project. Six people with learning disabilities made short videos to tell their story about working at the brewery.
- The research team looked at the stories. They found that working at the brewery helped people to feel more confident. They enjoyed learning new skills and doing good work. They felt proud of their work and as though they belonged at the brewery.
- Working at the brewery helped people learn life skills as well as brewing skills. More research would be needed to understand how this way of offering work can help more people.

## 1 Introduction

### 1.1 Employment for People With Intellectual Disabilities

Figures suggest that only 5%–6% of adults with intellectual disabilities (also termed ‘learning disabilities’ in the UK), are in employment (Department for Health and Social Care 2025) despite consistent findings emphasising a positive link between employment and quality of life for this population.^1^ Jahoda et al.'s (2008) review found that employment increased quality of life, well‐being and autonomy for people with intellectual disabilities. Subsequently, Jahoda et al.'s (2009) longitudinal study interviewed 35 people with intellectual disabilities as they started a new job, and again 9–12 months later. They found that work gave purpose, financial rewards, opportunities for relationships and increased self‐worth, but could also be a source of anxiety. If adaptations were not sufficient people worried about losing their jobs, and social relationships did not always leave people with a genuine sense of belonging. A review by Robertson et al. (2019) also concluded that employment was associated with better physical and mental health for this group, but the cross‐sectional nature of most studies made it difficult to infer causal links; those with better health may have been more likely to be employed. These studies suggest nuance is required to understand the potential benefits of work for people with intellectual disabilities.

Akkerman et al.'s (2018a, 2018b) study of job satisfaction in people with intellectual disabilities (*n* = 117) in the Netherlands attempted to understand these nuances with reference to self‐determination theory (Ryan and Deci 2000). They found that allowing workers to feel autonomous, effective and connected to others was more important to job satisfaction than their level of participation. They also found that conscientious workers tended to have their task demands increased, resulting in lower job satisfaction. Studies from other countries have also found that flexibility in job design, task matching and tailored support are crucial to the well‐being of people with intellectual disabilities in the workplace (see Flores et al. 2011; Meacham et al. 2017, 2021; Joyce et al. 2024).

Studies have also focused on different models of employment. Almalky's (2020) review differentiated between sheltered employment (i.e., exclusively for people with disabilities), supported employment (i.e., integrated with non‐disabled workers with support to develop skills), customised employment (i.e., supported within a competitive work setting) and competitive employment (i.e., equal wages to non‐disabled co‐workers). All forms had positive impacts on self‐esteem, self‐development, self‐confidence and independence, but benefits were more pronounced for supported or competitive employment. Randall et al. (2023) also reported similar findings. Almalky (2020) suggested that the integration opportunities in these models could enhance social skills and relationships. They also pointed out that sheltered employment could support people to learn skills that could lead to more integrated employment, and that there was a need to establish such developmental pathways.

Meltzer et al. (2018) reported similar findings in Australia comparing ‘open’ (i.e., mainstream, competitive) with ‘sheltered’ (i.e., segregated, production line type work) and social enterprise employment (i.e., organisations set up for community benefit employing marginalised groups). They found challenges to each type, with open employment often seen as less understanding. They suggested that social enterprises offered several benefits including community connection, supportive cultures and an emphasis on skill development, although there could be issues with waiting for paid positions, and there was not always sufficient focus on autonomy. Overall, it appears that the benefits from employment for people with intellectual disabilities relate strongly to the workplace set up.

### 1.2 Social Enterprise Brewery: The Community

The social enterprise brewery at the heart of this study was established in 2018 as an inclusive employment project for people with intellectual disabilities and autism. The majority of trainees have intellectual disabilities, although people with autism (without intellectual disabilities) were included in the research if they wished. The company brews, bottles, packs and sells beer, alongside a Tap Room service. In 2024, they provided 3300 h of volunteer work and over 700 h of work (paid and volunteering) for adults with intellectual disabilities and autism. At the time of the study there were five non‐disabled staff (two paid and three volunteers) who ran work sessions and twelve trainees (five in paid work and seven volunteers). The brewery also raises the profile of people with intellectual disabilities and autism within the local community.

The brewery combines elements of sheltered, supportive and competitive/open employment identified in the literature, within a social enterprise (Meltzer et al. 2018; Almalky 2020). Their developmental model matches tasks to people's abilities. People can start by volunteering with specific tasks (e.g., labelling and packing), sometimes as part of their support hours (i.e., perhaps closer to ‘sheltered employment’). Non‐disabled employees support trainees to develop their skills in aspects of brewing and customer service (supported employment) eventually leading to paid shifts which can include serving customers (i.e., closer to competitive employment).

As a social enterprise, the brewery wanted to capture the impact of their employment model. Previous attempts to do this had used questionnaire methodologies, but these were not sufficiently accessible and could not yield meaningful insights into trainees' lived experience. This limited the brewery's potential to demonstrate the broader value of its work, build social capital and develop future funding applications. This led the brewery board (represented by the third and fourth authors) to approach the researcher (first author and local resident) and request collaboration. This offered a unique opportunity to undertake real‐world research on the lived experiences of people with intellectual disabilities undertaking employment within a social enterprise that incorporated several employment models identified in the literature (e.g., Meltzer et al. 2018; Almalky 2020).

### 1.3 Community Based Participatory Research (CBPR)

Community Based Participatory Research (CBPR) is a framework where by research is developed in partnership with communities and grounded in the needs that they identify, rather than by academic researchers. Collins et al.'s. (2018) overview of CBPR within psychological research highlights its collaborative and flexible nature. Key characteristics of CBPR include: the importance of the relationships that communities develop with researchers; the central role of the community in developing the research questions; the flexibility required in research design to incorporate the community's needs and priorities; the role of community members in aspects of data collection or analysis; the importance of considering accessible and non‐academic formats for dissemination.

In this study, several elements aligned with a CBPR framework, most notably that the impetus for the research came from the brewery (i.e., the ‘community’), rather than the researcher, and that relationships and collaboration were crucial in developing the research questions, study design and execution.

This project brought together the community‐based, lived experience of adults with intellectual disabilities working at the brewery with academic and clinical expertise. The term ‘trainees’ will be used throughout this paper to represent this group as all roles at the brewery included a training element (i.e., including paid roles). The first author spent time with trainees and the brewery board (including the third and fourth authors) to develop the research question and design. These discussions identified that the research question needed to be sufficiently broad and accessible in order to explore trainees' experiences. The primary research question was ‘What is it like for people with intellectual disabilities to train and work at the brewery?’. A secondary aim was to explore the utility of the CPBR approach and data collection method with people with intellectual disabilities to inform future researchers.

## 2 Method

### 2.1 Design

The study was informed by a CBPR framework and funded by a scheme designed to build partnerships between community groups and university researchers.

After establishing the research question (see above), the first author visited the brewery over several weeks and joined social events held by trainees to develop the design. Discussions were held in small and large group and 1:1 formats to suit the needs of trainees. Time was taken to explain the concept of ‘research’ and working with the university. A qualitative design using digital storytelling as a data collection method was agreed. Digital stories are 3–5 min long and are a flexible method to allow people to tell their stories using multiple media sources such as voiceover, photographs, music, drawing and video clips. Digital storytelling developed as a way of empowering marginalised communities (Lambert 2013), and has been used as a data gathering method within participatory research, particularly with marginalised groups, such as Indigenous communities (Flicker and MacEntee 2020), women who have experienced relational abuse (Martin et al. 2019), people with intellectual disabilities (Harris et al. 2024) and a vulnerable marginalised community group (Voss et al., forthcoming). Fish and Syed (2021) provide a framework and guidelines for using digital storytelling within psychological research, arguing that this method offers greater potential to empower people to tell their unique stories compared to traditional interview methods.

Trainees were used to being photographed and videoed for local news items, and some trainees had produced artwork related to the brewery. As a group they were more enthused by this method of data collection and felt it was more accessible than traditional interviews. This method also had the potential to facilitate the creation of material that the brewery could use to communicate their impact more effectively to funders and stakeholders.

### 2.2 Ethics

Ethical approval was granted by the University of Plymouth Faculty of Health Ethics Committee. All study documents were drafted and discussed with trainees to ensure accessibility. Additional documents enabled participants to gain consent from people if they wished to include an image of them in their digital stories (e.g., colleagues). Digital stories could potentially identify participants, and this was explicitly discussed when establishing consent. Participants had experience of choosing whether to be included in local news items about the brewery, or on social media, and were therefore able to understand the implications of their digital story being identifiable. Participants were advised that they did not need to include personal information (e.g., such as pictures of themselves) in their stories. Different levels of information sharing were also offered as part of the consent process (see Table 1). These options were discussed thoroughly with participants using accessible information, and they were reminded of these when creating their digital story, and once the stories were complete, given options to change their mind. The Mental Capacity Act (2008) guided decisions about whether participants had capacity to consent. Potential participants were taken slowly through the accessible information materials and asked to explain each point in their own words. Breaks were given where needed. Any trainee who struggled to understand, retain, weigh up or communicate key information about the study was thanked for their time and their participation was not pursued.

**TABLE 1:** Levels of consent given as options for sharing digital stories.

	Option for sharing digital story	Implication discussed with potential participants
Level 1	Used by University researchers (if you wish to keep your story completely private then only tick this box)	This would mean that no one else apart from the researchers would see the digital story, unless you choose to share it. This keeps your story private.
Level 2	Shared with the Brewing Company (they may use it for their website but they will always ask you first)	The Brewery can see your story. They may want to use it on their website which means people will know who you are. They will always ask you first.
Level 3	Used as an example in the report or at a conference presentation (if you have personal details in your story this means these will no longer be private).	Full sharing option. This means that the researchers can include your story in their report or when giving a presentation. This means other people may see your story and know who you are.

### 2.3 Participants

A poster advertising the research was displayed in the brewery. Trainees who expressed an interest were visited at work by the second author who discussed participation using accessible documents. At the time of the study there were 12 trainees at the brewery. The research was discussed with eleven trainees, with seven expressing an interest. One of the trainees was found not to have capacity to consent to the research (see ‘ethics’ above). The remaining six had capacity to give informed consent to take part. A digital storytelling workshop was arranged and five participants attended. The sixth participant was unable to attend on the day but still wished to take part, so the second author supported them to make a digital story on a 1:1 basis at the brewery. Basic demographic information was collected from participants, including asking them what term they used to describe their disabilities (see Table 2). Participants all used UK terminology and self‐reported having ‘learning disabilities’.^2^ Pseudonyms are used in this paper, although the digital stories linked below (see Findings) are not anonymised. They are shared in accordance with the choices made by participants during the consent process (see Table 1).

**TABLE 2:** Demographic information about participants (self‐reported).

Pseudonym	Gender	Age	Length of time working at brewery (years)	Type of disabilities	Other health issues
Daniel	Male	32	5	Learning disabilities	
Mark	Male	29	3	Learning disabilities	
Steve	Male	57	2	Learning disabilities	None
Robert	Male	60	5.5	Learning disabilities Autism	Heart issues
Harry	Male	34	3	Learning disabilities Autism	ADHD dysphasia
Lucy	Female	24	5	Learning disabilities Hearing loss	

### 2.4 Procedure

The digital story workshop was conducted in a local venue separate from the brewery. Each participant was supplied with a laptop borrowed from the university and a log in for basic video creation software. Participants were reminded about the purpose of the study and that they could pause or stop their participation at any time. Participants were supported as required by a combination of the researchers, brewery staff and their own support workers to develop a story about their experience at the brewery using the prompts ‘tell us about your work at the brewery’ and ‘how does working at the brewery make you feel?’ and ‘what are the good things and bad things about working at the brewery?’ These stories were transcribed live by a supporter who regularly checked to ensure that they had captured the intended meaning. The process of the workshop is illustrated in Figure 1.

**FIGURE 1:**
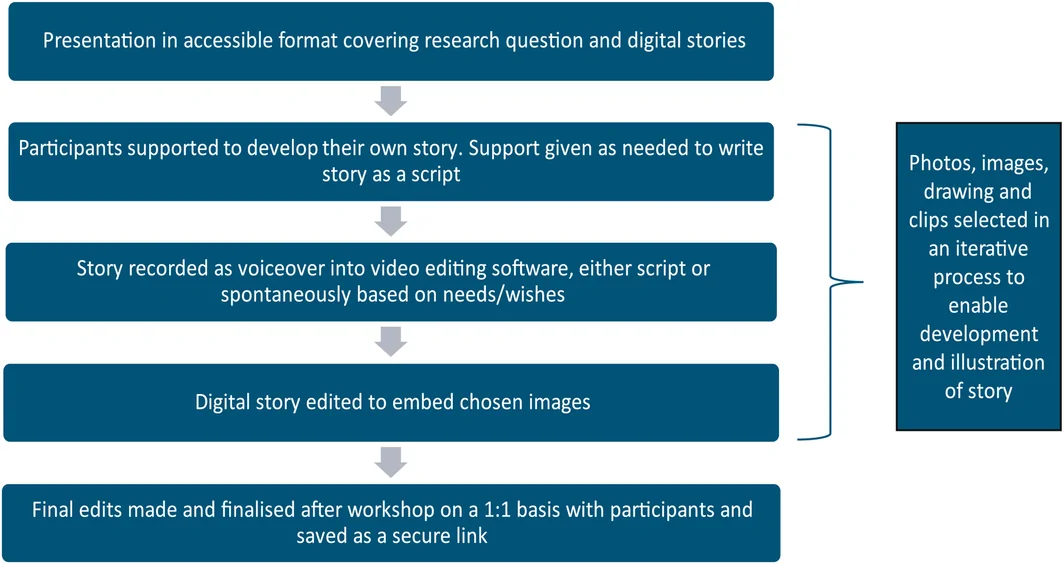
Digital story workshop process.

Participants selected photos and other images throughout the digital storytelling workshop. For some participants, a specific element of their story prompted a search for a corresponding image. For others, a favoured image prompted them to relay a specific element of their story. Two participants used their own drawings, which were scanned in. Participants who could read generally preferred to read their story from a script when recording their voiceover. Participants who were less confident with reading or speaking aloud were reminded about the key elements of their story prior to recording the voiceover, and they then produced a spontaneous version. No correction was offered if these participants deviated from their original story; both versions were used for analysis. Voiceovers were played back to participants to ensure that they were happy with them before proceeding. Support was required for the video editing process, as this proved complicated for several trainees. This was conducted using person‐centred principles to ensure that edits reflected the wishes of participants. Time demands meant that final edits were completed after the workshop, and finalised in 1:1 sessions with participants. Once stories were finalised, participants were given a secure link to view and share it as they wished. Each story voiceover was transcribed anonymously by the second author, and these were saved securely alongside written versions.

### 2.5 Positionality

This study adopted a critical realist perspective (Bhaskar 1975) because this allowed acknowledgement of participants' realities of training and working at the brewery, but at the same time allowed interpretation of the ways in which their experience of work could be shaped by their cultural context and broader social structures. The researchers discussed their own assumptions and position in relation to the topic prior to conducting the analysis. The first author had nearly two decades of experience working as a clinical psychologist in services for people with intellectual disabilities, and lived in the community where the brewery was based. The second author had experience as a support worker, had undertaken undergraduate research with people with intellectual disabilities, and was also a community resident. Both agreed that their experiences had led them to believe that opportunities for valued and meaningful activity were important for the well‐being of people with intellectual disabilities, and that as local residents, the brewery was popular in the community. The potential for these beliefs to shape the research was actively discussed during analysis, and journaling was used to reflect on the coding process in relation to researchers' beliefs.

### 2.6 Analysis

There are numerous ways to analyse data from digital stories (Fish and Syed 2021). Reflexive thematic analysis (Braun and Clarke 2022) was chosen for its ability to be flexible to a range of data sources (i.e., including visual data in addition to transcripts), and because it is considered to fit within a critical realist epistemology. Transcripts included both the written version of the story and the voiceover as there were some differences between these for some participants (see ‘Procedure’). This ensured that all information given was captured. Digital stories were viewed several times during analysis to capture meaning of the chosen images alongside the voiceover.

Story transcripts and images were analysed in six stages (Braun and Clarke 2022): (i) familiarisation with the data set; (ii) coding; (iii) generating initial themes; (iv) developing and reviewing themes; (v) refining, defining and naming themes, (vi) writing up. Stages (i‐iii) were conducted by the first and third authors and facilitated by memos, reflective journaling and discussion. Coding was inductive (i.e., data rather than theory driven) and included both semantic (i.e., surface) and latent (i.e., implicit) meanings in the data. From stage iv, the analysis was developed in consultation with participants and non‐disabled brewery staff (including the third and fourth authors), in a ‘back and forth’ process with the data set to ensure that the themes captured the essence of the data and made sense to participants. A draft thematic map was presented and discussed with participants, alongside visuals to illustrate themes labels where possible. This led to further refinements (e.g., changing theme labels to ensure accessibility, clarifying subthemes). The overarching theme was developed at this stage, as it was repeatedly mentioned by stakeholders when discussing the data. Participants were invited to appear in a short video to disseminate the findings. Four participants took part, all of whom spontaneously spoke about the themes when discussing their brewery work (Burt 2025).

## 3 Findings

Six digital stories were completed. All six participants consented to their digital stories being used by the brewery for promotion purposes (i.e., Level 2 in Table 1, above). Four participants consented to their digital stories being used in academic dissemination such as journal papers and conference presentations (i.e., Level 3 in Table 1, above). The links to these four stories are below:

- https://youtu.be/kXNYvYpBRCs.
- https://youtu.be/71zKwQhJxt0.
- https://youtu.be/hTV8D8qT9uY.
- https://youtu.be/WAN4G6K2I1Y.

The analysis led to the development of three main themes, each with subthemes to enable a deeper understanding (see Figure 2). An overarching theme ‘I feel more confident’ connected the themes. Themes will be discussed as they are presented, with a more general discussion below.

**FIGURE 2:**
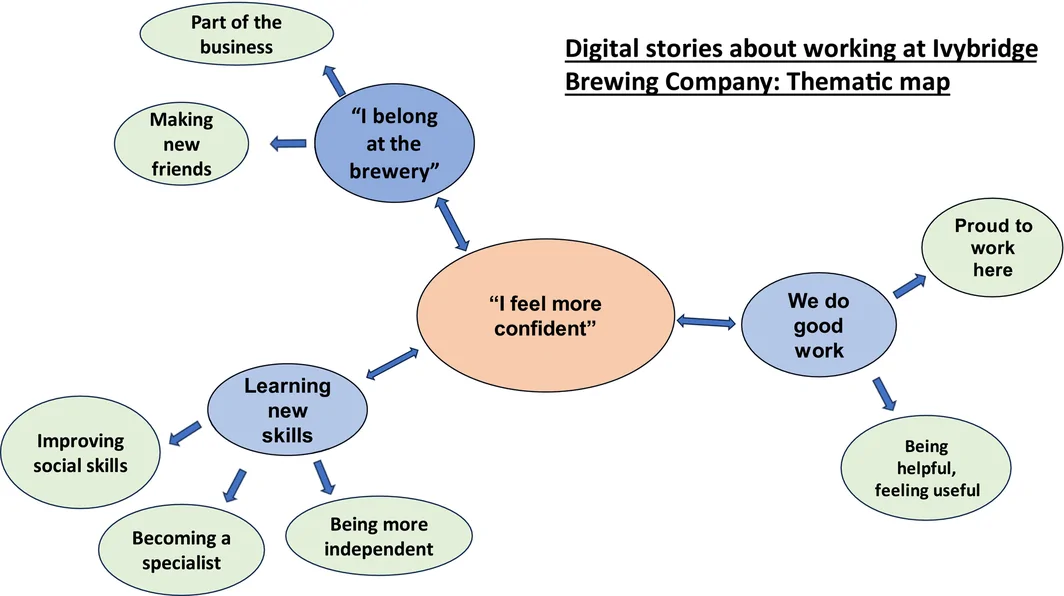
Thematic map of digital story transcripts.

### 3.1 Theme 1. Learning New Skills

Participants spoke in detail about learning new skills, many of which were related to the brewing process and running the tap room. Participants chose images for their digital stories depicting these skills (e.g., picking hops, bottling, brewing, serving customers).

Daniel: Whilst at the brewery I cleaned the lines out. In the tap room, I serve the customers, tell them how much they owe, and take card payments.

The data further suggested that participants' learning went beyond skills specific to running a brewery. Three subthemes were developed to capture the breadth of this learning.

#### 3.1.1 Subtheme 1: Improving Social Skills

Participants spoke about the development of their ability to talk and interact with others, and to get along with people in the workplace

Mark: I'm better at serving customers now because I'm better at talking to people now.

Daniel: Working at the [brewery] has … helped me with my skills, listening to what customers want and talking to them…… […]…it has not only helped with learning brewing skills but also helped me with my social skills… I used to do the bottling and capping of the beers but I realised that I didn't enjoy this task. This was more about learning who I worked well with and who I didn't […] This helped me to learn it's okay not to like things or people but I should always be kind and polite.

Daniel's quote describes broader learning about respectful relationships at work; professional skills which could potentially be transferable to other settings.

#### 3.1.2 Subtheme 2: Becoming a Specialist

Participants spoke at length about their knowledge of the brewing process.

Mark: I know a lot more about beer. Hops are the plant we use. We get them from the USA. Citrus hop is one. My favourite is Amarillo. I watch YouTube videos about brewing beer. I like to see if they do it the same way and I learn more.

Harry: In my time at the Brewery I've learned about different beers being made, how it's made and how to produce the beer, cleaning and labelling beer bottles, stock taking. I also learnt to brew, putting the grains in the bub, boiling wort, cleaning the mash tub once the strains have broken down.

There was also data to suggest that the work had enabled participants to develop other specialist skills.

Harry: I will be doing IT courses which will potentially gain me another job at the brewery with stocktaking on the computer.

The ‘special interests’ of people with intellectual disabilities and/or autism can be idiosyncratic and not always valued by wider society. This data suggests that work can create an opportunity for people to develop specialist knowledge that has value for the wider community. This led to a discussion amongst the researchers about how all of us had interests that we were able to channel via our employment which afforded us social status as ‘experts’, and were not given the arguably more patronising term ‘special interests’. We reflected on the importance of work allowing people with intellectual disabilities the opportunities to become valued experts.

#### 3.1.3 Subtheme 3: Becoming Independent

Participants made links between their work and greater independence. Mark's quote below revealed a shift towards experiencing himself more as an adult.

Mark: I came with my enabler first but now I come on my own… I was working with my mum a few years ago but I prefer working at the brewery because I'm more independent. It makes me feel more grown up. It's a good thing for me… Simon used to tell my mum about the time of sessions and now he tells me because I work for him not my mum and I'm better at remembering.

Daniel chose to use images of himself in his own home in his story to represent how his increased independence had led to a reduction in his support hours. Getting paid also featured, as for some participants this marked a shift in their independence and access to other opportunities.

Daniel: Since working at [the brewery] I have moved out of home. I live independently. I don't need as much support now. I started to get paid helps me be independent.

Not all participants were paid for their work, which Robert referred to.

Robert: Some places you get paid, I don't, I feel alright about it, happy to not get paid.

Although he appeared happy, it was clearly on his mind and one could speculate whether this reflected acquiescence at some level.

### 3.2 Theme 2: We Do Good Work

This theme reflected the participants' stories of doing work that they enjoyed, and that was valuable to others. Notice this in Robert's extract below:

Robert: Cleaning the bottles, I feel good, it's good fun. I clean the floor, I clean the mess up, I like it.

Two subthemes were developed to represent different aspects of the ‘good work’ spoken about in the digital stories.

#### 3.2.1 Subtheme 1: Being Helpful and Feeling Useful

Participants' sense of their work being valued as ‘good’ was reflected in data that referenced being helpful or feeling useful. In particular, the data suggested that interactions with customers enabled participants to experience themselves as helpful.

Robert: I like serving customers and making the Brewery money… They make me feel alright; I like talking to the customers.

Being helpful appeared linked to participants experiencing themselves as experts and essential workers, potentially shifting power dynamics between those with and without intellectual disabilities.

Daniel: I know what beers people like.

Mark: I enjoy working with the customers‐ finding out what beers they like.

Lucy: I could come into work because we were an essential service. I felt happy as we were helping people.

Several participants gave broader examples of feeling useful, enabling a deeper understanding of how they experienced value in their work. For example, Steve linked his skills to the running of the brewery and referenced the overarching theme.

Steve: I was helping the plumber with the tiles and the electrician with the wires…. I carry big sacks of wheat, it makes me feel confident when helping.

‘Feeling useful’ was also interpreted through data reflecting a sense of purpose in being active and engaged in work, which appeared to link to aspects of well‐being.

Mark: If I didn't work here I'd be doing nothing and I'd be annoyed.

Steve: It is nice to be here, it gets me out of the house instead of getting bored.

Daniel: It has helped me to have purpose in getting up, getting showered and getting out the door.

#### 3.2.2 Subtheme 2: Proud to Work Here

This subtheme captured data that appeared to reflect the pride that participants had about their work, despite ‘proud’ or ‘pride’ not being explicitly named

Robert: The brewery is my work, I like telling people, I tell people all the time…. I like wearing the uniform to let people see.

Robert took pride in his uniform, possibly because it reinforced his identity as a worker in a valued local business. Robert was also a keen artist whose drawings had been displayed inside the brewery and used as label designs. He included his drawings in his digital story; one was a colourful scene of people, including himself, inside the brewery, which was interpreted as reflecting his pride in working in a busy and successful place.

Pride in the work was also interpreted when participants used images of the product in their stories, referenced winning awards, and compared their product to others

Lucy: I have been to the food and drink devon awards […], we won gold, silver and platinum awards.

Daniel: We even got an award for good service and for good beer this last week.

Mark: We made a beer with [neighbouring brewery]. Our beer is better.

### 3.3 Theme 3: I Belong at the Brewing Company

This theme captured participants' stories of the brewery as a place where they belonged and felt accepted.

Daniel: I feel that I belong at [brewery]. I am made to feel comfortable for being who I am.

There was acknowledgement of the brewery as being a place that specifically focused on people who had what Harry termed ‘learning differences’.

Harry: It's accepting, patient, accommodating to people with learning differences. In my case, the tasks I do don't cause too much stress; I'm able to work at my own strength.

#### 3.3.1 Subtheme 1: Part of the Business

Participants appeared to feel that they were part of the business. It was not simply where they worked; they were invested in the product and the future of the brewery. Several participants spoke about plans for the expansion of the tap room, which were prominent in their minds at the time of data collection.

Harry: It will be a place where it provides more opportunities, and more space will be made for the taproom and store

Mark: I'm looking forward to moving into next door, we'll have more room for more customers…. I'm going to do a beer fridge in the new place to keep the beer cold.

Steve incorporated drawings into his digital story that he had made planning improvements to the tap room.

Steve: I came up with an idea for new counters for next door in my drawings… I am happy to be more involved in building… Looking forward to getting this place open.

These extracts reflect a sense of feeling actively involved in brewery developments, with opportunities for trainees to use their new skills to make their role meaningful for the business.

#### 3.3.2 Subtheme 2: Making New Friends

Many participants spoke about friendships with work colleagues and how these were important to their sense of belonging in the brewery. Several participants chose images that included their colleagues, either during work or at social events. This was distinct from the ‘social skills’ subtheme as the focus here was on personal relationships rather than professional skills.

Steve: I have a laugh and I have made friends… Having social evenings‐nice to be together and have tea together.

Lucy: I like working at the brewery because I meet other people and make friends.

Daniel: It has helped my confidence to be able to meet new people and talk to them; some have now become my mates.

Notice that Daniel's quote also links to the overarching theme below.

### 3.4 Overarching Theme: ‘I Feel More Confident’

Confidence formed an initial code, but as analysis developed alongside stakeholders, it became apparent that this underpinned much of the data and linked themes together, so it was eventually positioned as an overarching theme. Participants spoke both explicitly and implicitly about gaining confidence from working at the brewery, in terms of their learning and skills (theme 1), the sense that they were doing good work (theme 2), and their sense of belonging (theme 3).

Lucy: I feel confident and happy to work there as I've been there right from the beginning, even though I don't like beer!

Steve: It has made me confident mixing in…. working with different people and doing something different….it makes me feel confident when helping

During informal discussions with trainees and family members, confidence was regularly mentioned as a key difference in people's lives since working at the brewery. It was also central to how participants spoke when creating a dissemination video for the research. Arts based activities were facilitated in the brewery with trainees between data collection and analysis. This was unrelated to the current project but the outputs resonated strongly with the analysis, including words such as ‘confident’, ‘friend’ and ‘learn’ (see Figure 3).

**FIGURE 3:**
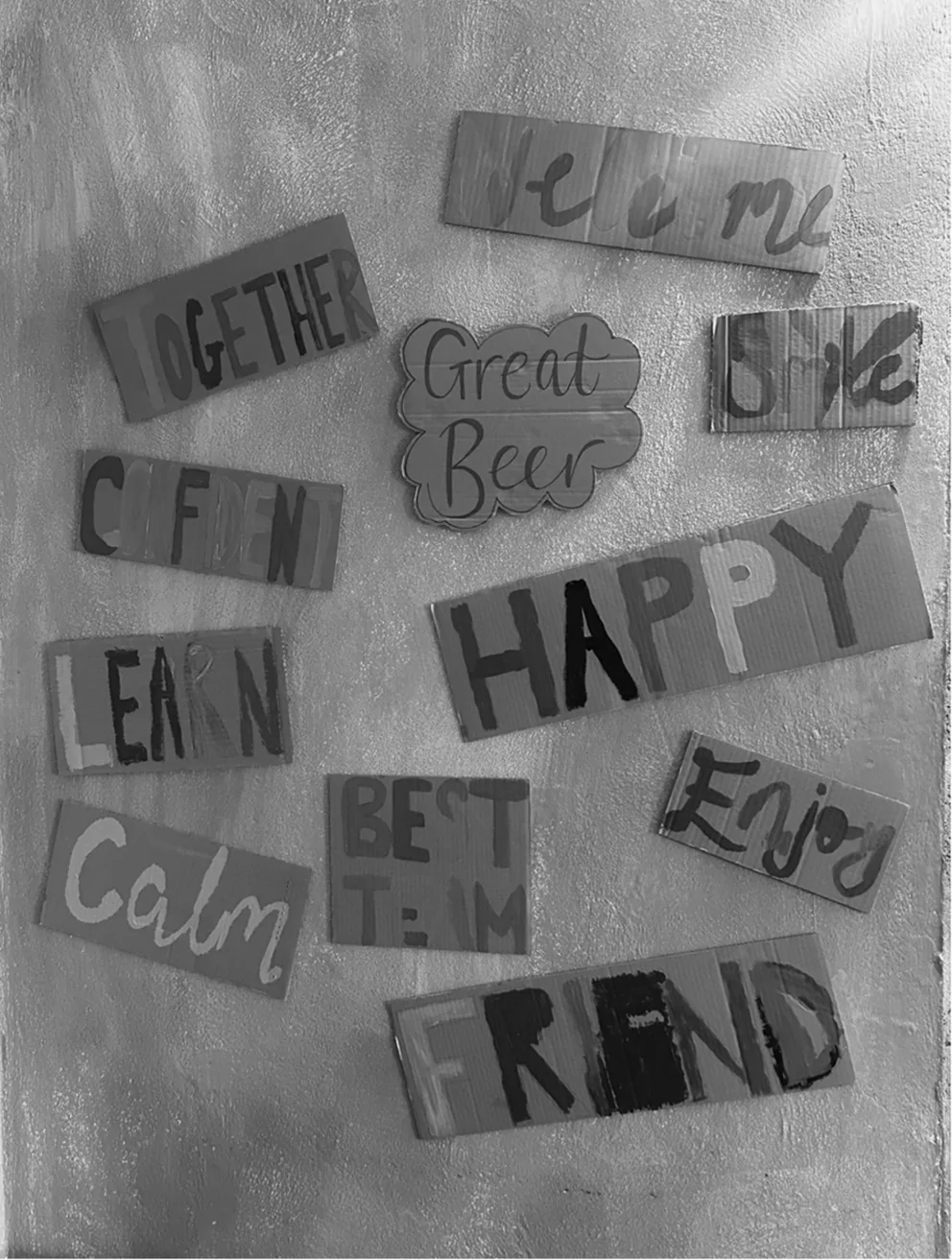
Artwork created at the brewery following the project.

## 4 General Discussion

This study was informed by a CBPR framework, and explored the experiences of a small sample of people with intellectual disabilities (and autism) working in a social enterprise brewery. The findings support existing literature on the positive benefits of employment for this group (e.g., Jahoda et al. 2008, 2009; Robertson et al. 2019). Participants created stories about the skills they had learned, feeling proud of the work and as though they belonged at the brewery. Linking these themes was a consistent story of how working at the brewery had made them feel more confident.

The findings also link to those of Akkerman et al. (2018a) in relation to self‐determination theory (Ryan and Deci 2000), specifically the importance of feeling competent and autonomous. Garrels and Arvidsson (2019) reported that self‐determination can be lower in people with intellectual disabilities, and suggested that it can be enhanced when learning is ‘scaffolded’ to match the person's needs. This could be linked to the brewery model, which uniquely emphasised development from volunteering to paid work, appearing to shift flexibly between elements of sheltered, supportive, customised and competitive employment identified by Almalky (2020), within the benefits of a social enterprise as identified by Meltzer et al. (2018). This also supports Joyce et al. (2024) who found that diversity of roles, customised training and task matching were important in supporting the well‐being of people with intellectual disabilities at work. Although causal links are not possible from this study, it could be hypothesised that the brewery's developmental, flexible and person‐centred approach acted to ‘scaffold’ trainees towards greater self‐determination. This would need to be explored in more detail in future studies, utilising specific measures (e.g., as proposed by Verdugo et al. 2023) and a longitudinal design. With Robert's comments about not being paid in mind, it may also be helpful to understand how different skill levels between trainees are best managed within this model.

The sample in this study was predominantly male. Females are in the minority at the brewery and only one participated in the study. There is a gender gap in employment for people with intellectual disabilities, with females much less likely to be employed (Nuffield Trust 2023). The reasons for this are not clear but could reflect additional disadvantage for women with intellectual disabilities. Brewing can tend to be a stereotypically ‘male’ pursuit, so this specific workplace may have unintentionally limited female employment. More generally, the relative disadvantage of women with intellectual disabilities in accessing employment should be a focus of future research efforts with a broader population.

The study is limited in terms of size and breadth, but this needs to be offset with the advantages of partnering with a community and researching the topics that matter to them, which can strengthen local impact. It was possible to apply a CBPR framework and engage people with intellectual disabilities in the research process with accessible communication materials and repeated visits to build relationships and understanding. Participants have since visited the University to talk to students about employment, something that may not have been possible prior to this project. This suggests that co‐produced research is feasible with this group, and that the benefits can extend beyond the life of the project.

Digital storytelling proved to be flexible and accessible as a data collection method, allowing participants to combine their own words with chosen images and to create a final video that they could keep, and that the brewery was able to use (with permission). Close attention to person‐centred practise was vital to ensure that the final video edits conformed to the participants' wishes. The researchers would advocate for the use of this method in future research, but with some caution as considerable support was required for participants to operate the software. Researchers would therefore be well advised to consult with participants as to their existing skills to build in sufficient support, adaptations and time in their research plan.

The brewery in this study affords opportunities for the local community to engage with people with intellectual disabilities. The contact hypothesis (Allport 1954) suggests that exposure is key to reducing stigma, which can be high towards people with intellectual disabilities and autism (Pelleboer‐Gunnink et al. 2019). Meltzer et al. (2018) identified community connection as a benefit of social enterprise employment for people with intellectual disabilities, but this potential was not fully explored in this study. Future studies on community attitudes towards employing people with intellectual disabilities could enable the brewery to evidence their broader social impact.

To conclude, this small, community‐based study used a novel participatory method to find that people with intellectual disabilities could identify immediate and broader benefits of their training and employment within a social enterprise brewery. Combining different models of employment support within one setting and moving flexibly between them appears to have potential in terms of developing people's skills and confidence. We also demonstrated the potential of the CBPR framework and specific digital storytelling method for this group. Further research would be needed to better understand the impact of this flexible employment model on self‐determination and its broader community impact.

## Funding

This work was supported by the Get Involved Awards scheme at the University of Plymouth (grant no. GH105317‐104).

## Conflicts of Interest

The authors note that Simon Rundle is CEO of the brewery in question. As a social enterprise, any profits that the brewery makes are put back into the business, so he does not personally profit from his role. Karen Gresty is a volunteer with the brewery. Both Simon and Karen are retired academics, formerly employed at the University of Plymouth in a discipline unrelated the topic (marine biology). Given the community based participatory design of the project, co‐authorship felt important given that both Simon and Karen provided impetus and were co‐applicants for the bid that funded the project. Neither Simon nor Karen analysed the data, as it was outside of their expertise and there was the potential for a conflicts of interest given their knowledge of the participants. They did provide feedback on the findings and ensured that details were accurate in the writing of this paper. Co‐authorship is intended to represent the collaborative nature of the project, and is common for community based participatory designs.

## Data Availability

The data that support the findings of this study are available on request from the corresponding author. The data are not publicly available due to privacy or ethical restrictions.

## Endnotes

^1^
The lay summary was written with the support of participants who preferred the term ‘learning disabilities’ as they do not use the term ‘intellectual disabilities’.

^2^
One participant was considered by researchers as predominantly autistic and less likely to have met standard criteria for intellectual disabilities, but given the CPBR design, it was felt inappropriate to contradict their self‐report.
